# Hospital Meetings

**Published:** 1906-06-09

**Authors:** 


### HOSPITAL MEETINGS.

### MIDDLESEX HOSPITAL.

A quarterly meeting of the Court of Governors of the Middlesex Hospital was held on May 31st, Prince Francis of Teck presiding.

Major-General Lord Cheylesmore, the Chairman of the Weekly Board, moved the adoption of the report presented, which stated that in succession to Sir Arthur Watson, K.C., who on account of failing health had withdrawn from active participation in the administration of the hospital, H.S.H. Prince Francis of Teck, a Vice-President, had been nominated Deputy-Chairman of the Weekly Board, and that Mr. Reginald Lucas had been elected to fill a vacancy on the Board. Prince Francis of Teck had issued 25,000 copies of an appeal for support to the inhabitants of Marylebone, on the ground that 45 per cent. of the out-patients and a large proportion of the in-patients are dwellers in the borough. The appeal had in some degree augmented the list of subscribers, but many who did not hesitate to avail themselves of the charity for their servants and employés were yet tardy in recognising their responsibility in the matter of contributing to its maintenance. With the object of raising £20,000 to meet the estimated deficiency for the current year, arrangements had been made to hold a festival dinner in the Mercers' Hall on June 12, under the presidency of the Duke of Connaught, but His Royal Highness's recent family bereavement having necessitated the cancelling of all his engagements, it had been decided to postpone the function. In these circumstances Mr. H. L. Bischoffsheim, to whose liberality the institution was already much indebted, had contributed £5,000 to repay advances from the bankers and meet other immediate expenses, so lightening the loss entailed by the postponement.

The new methods of advertising, which took the form of literary articles published in the daily papers upon the work performed by the hospital in the alleviation of sickness and disease, and accentuating the urgent need for increased pecuniary assistance, had been found of much greater value than the formal notifications it was customary to insert in the past. The Board had, in response to an invitation by the Earl of Lytton, agreed to co-operate with a scheme for the establishment of a home of recovery, intended for the reception and accommodation during convalescence of poor persons suffering from wounds, the result of accident or operation, and still requiring surgical supervision. The chief work of the cancer research laboratories during the quarter had been the compilation of the record of work done in 1905, which would be issued immediately. Beside the usual short details of the patients under treatment for malignant disease during the year, it would contain fifteen pages of considerable importance. These cancer reports were gaining credit among workers on the subject, and requests for copies came from all parts of the world. The convalescent home had done satisfactory work, its benefits having been extended to 264 cases during the quarter. In the electrical department the most gratifying results continued to be obtained. During the quarter 1,184 in-patients were treated and 10,853 out-patients, with 27,338 attendances—an average of 300 daily.

Mr. Andrew Clark seconded the adoption of the report, emphasising the great usefulness of the electrical department. The motion was agreed to.

### NORTH-EASTERN HOSPITAL FOR CHILDREN.

Sir Walter Johnson took the chair at the annual meeting of the Governors of this hospital on May 31.

In moving the adoption of the report the Chairman remarked that the increase in the number of in-patients from 1,258 in 1904 to 1,609, and the large increase in the number of attendances, showed the growing needs of the hospital, and was a justification for the enlargements undertaken by the committee. The nurses' home and laundry were put in hand in July last year, and the former was just ready for the reception of the nurses and the laundry would be completed shortly. The Lord Mayor and Lady Mayoress had promised to come in State and open these new buildings on July 2. A festival dinner had been held, which resulted in the receipt of something under £3,000. The luncheon held in the previous year had resulted in £4,656 having been added to the funds, so that they were £1,600 short this year. Greater efforts would have to be put forth to make up this deficiency. In order to show their grateful appreciation of the interest taken in the work of the hospital by Princess Henry of Battenberg, the committee had that day telegraphed their congratulations to Her Royal Highness, and also to Princess Ena on the occasion of her wedding day, expressing the good wishes of the North-Eastern Hospital. They were happy in possessing a most efficient staff, who all, from the highest to the lowest, did their work exceedingly well. The total expenditure for 1905 was £11,428, and the receipts £10,467. The building fund at the end of the year showed an indebtedness of £8,200, and it was a matter for great regret to the committee that they had not been able to make any further reduction in this liability. A special grant of £500 had been received from King Edward's Hospital Fund towards reducing this debt. Mr. Abram Lyle, a member of the committee, had given a very generous contribution of £500 towards the cost of the nurses' home. During the year the Editor of *Little Folks* had handed to the hospital sums amounting to £630, thus completing the amount of £3,000 which he had undertaken to collect from his readers for the naming of a ward. The Editor had also generously entered upon a new scheme for establishing a *Little Folks* Home as a country branch and convalescent home for the hospital. This would be an immense boon to their patients. The enlargement of the hospital, which had more than doubled the bed accommodation, had necessarily increased the maintenance expenditure. Their assured income was only £250 from invested property; while they received £700 from patients' fees, subscriptions £2,200, the various hospital funds contributed £1,100, collecting boxes £200, and, since the expenditure was £11,000, they had thus £6,500 to raise in the best manner possible. More help was needed to enable the hospital to keep in full and efficient work.

The resolution was seconded by Mr. Charles Port.

The Rev. W. G. Morcom, hon. chaplain, in proposing a vote of thanks to the staff, alluded to a recent attack that had been made upon nurses in a lecture delivered by a lady, and indignantly repudiated, on behalf of the nurses of the North-Eastern Hospital, the charges therein brought.

### INVALID CHILDREN'S AID ASSOCIATION.

The annual meeting of this Association was held at 17 Bruton Street on May 30, by kind permission of Lord and Lady Stratheden and Campbell. There was a very large attendance and the chair was taken by Sir Frederick Treves, who, in his opening speech, said that the Asscoiation aimed at visiting invalid and crippled children and looking after them in every possible way; in watching over them from the beginning of their trouble to the end; in fact, in seeing them out of the wood. It professed to mother these children, to become their *alma mater*, certainly no mother ever had such a family of cripples, who were fostered and looked after by this Society, in its capacity of foster-mother. Many children suffering from crippled limbs might have been cured if they had been treated early enough. The indifference of the mothers of these poor children was extraordinary; they would allow a child to go on without treatment until the limb became dislocated by disease. The melancholy feature of hospital work was that as soon as a child patient could be moved it had to go out. This was on account of the crowds of people clamouring to come in. Convalescence in the East End was a monstrous perversion of terms, nothing more hideous than some forms of it could be imagined. Then came in the magnificent work of the Association, of which he could not speak in too enthusiastic terms.

The Bishop of London moved the adoption of the report, and thanked the Chairman for the most touching address he had yet heard. It was not always that one of the most gifted surgeons in the world had also the gift of oratory. He himself had worked for nine years, visiting day by day in the slums of East London, and the very name of the Association brought back pictures of scenes that were never to be forgotten. He had lately entertained the "Guild of the Brave Poor Things," whose founder was now in the room. About thirty boys came, not one of whom had his proper allowance of limbs, yet all of them, thanks to the kind influence they were under, were brave and cheerful. He could corroborate what their Chairman had said as to the way children were hustled out of the hospitals to make room for others. It was one of the cruellest things in life for those who performed operations to see cases saved by their skill perishing for want of sufficient fresh air, good food, or a surgical instrument, for want of a helping hand. It was exactly these cases that this Association helped. There was no cause he could plead for with a better conscience than this, and he pleaded for very liberal subscriptions and also for more personal service, especially from young girls.

Dr. Hutchison seconded the motion, and said that, speaking from the point of view of a doctor, this Association might be called the Helpless Doctors' Aid Association, as it stepped in and helped the doctors. The most harassing thing about hospital work was the feeling that they could not do for patients what they would like to do, through inability to get their treatment carried out, and here the Association stepped in.

Mrs. Craigie proposed a resolution approving of the work, and remarked that 12,000 children had received relief through this Association, but this was but a small number in proportion to what they wished to do. A crippled child was a constant care, help once begun must be continued to the end.

Mr. Harold Spender, in seconding the resolution, said the committee were anxious, not only to look after the child while sick and ailing, but to see that it was well started in life by means of some system of apprenticeship. For this scheme more funds would be needed in addition to their ordinary requirements.

Votes of thanks to the Chairman and to Lord and Lady Stratheden and Campbell were subsequently passed

